# Supplementary material for: Structure-based discovery of inhibitors of Mac1 domain of nonstructural protein-3 of SARS-CoV-2 by machine learning-augmented screening of chemical space
Source: bioRxiv. 2025 Sep 9:2025.09.05.674529. Preprint. [Version 2] doi: 10.1101/2025.09.05.674529 (PMC12440007; doi:10.1101/2025.09.05.674529)
Supplement: Supplement 1 [file media-1.docx]

**Supplementary Material - Structure-based discovery of inhibitors of Mac1 domain of nonstructural protein-3 of SARS-CoV-2 by machine learning-augmented screening of chemical space**

Fuqiang Ban^1,#^, Rahul Ravichandran^2,#^, Galen J. Correy^3^, Oleksandra Herasymenko^4^, Madhushika Silva^4^, Suzanne Ackloo^4^, Albina Bolotokova^4^, Irene Chau^4^, Elisa Gibson^4^, Rachel Harding^4,6,7^, Ashley Hutchinson^4^, Peter Loppnau^4^, James S. Fraser^3^, Matthieu Schapira^4,5,6^, Artem Cherkasov^1,^*, Francesco Gentile^2,8,^*

*^1^The Vancouver Prostate Centre and Department of Urologic Sciences, University of British Columbia, Vancouver, BC, Canada*

*^2^Department of Chemistry and Biomolecular Sciences, University of Ottawa, Ottawa, ON, Canada*

*^3^Department of Bioengineering and Therapeutic Sciences, University of California, San Francisco, San Francisco, CA, United States*

*^4^Structural Genomics Consortium, University of Toronto, Toronto, ON, Canada*

*^5^Princess Margaret Cancer Centre, University Health Network, Toronto, ON, Canada*

*^6^Department of Pharmacology & Toxicology, University of Toronto, Toronto, ON, Canada*

*^7^Leslie Dan Faculty of Pharmacy, University of Toronto, Toronto, ON, Canada*

*^8^Ottawa Institute of Systems Biology, Ottawa, ON, Canada*

*^#^Equal contribution*

**Emails:* [*acherkasov@prostatecentre.com*](mailto:acherkasov@prostatecentre.com) *(AC),* [*fgentile@uottawa.ca*](mailto:fgentile@uottawa.ca) *(FG)*

**Supplementary Figures**

**
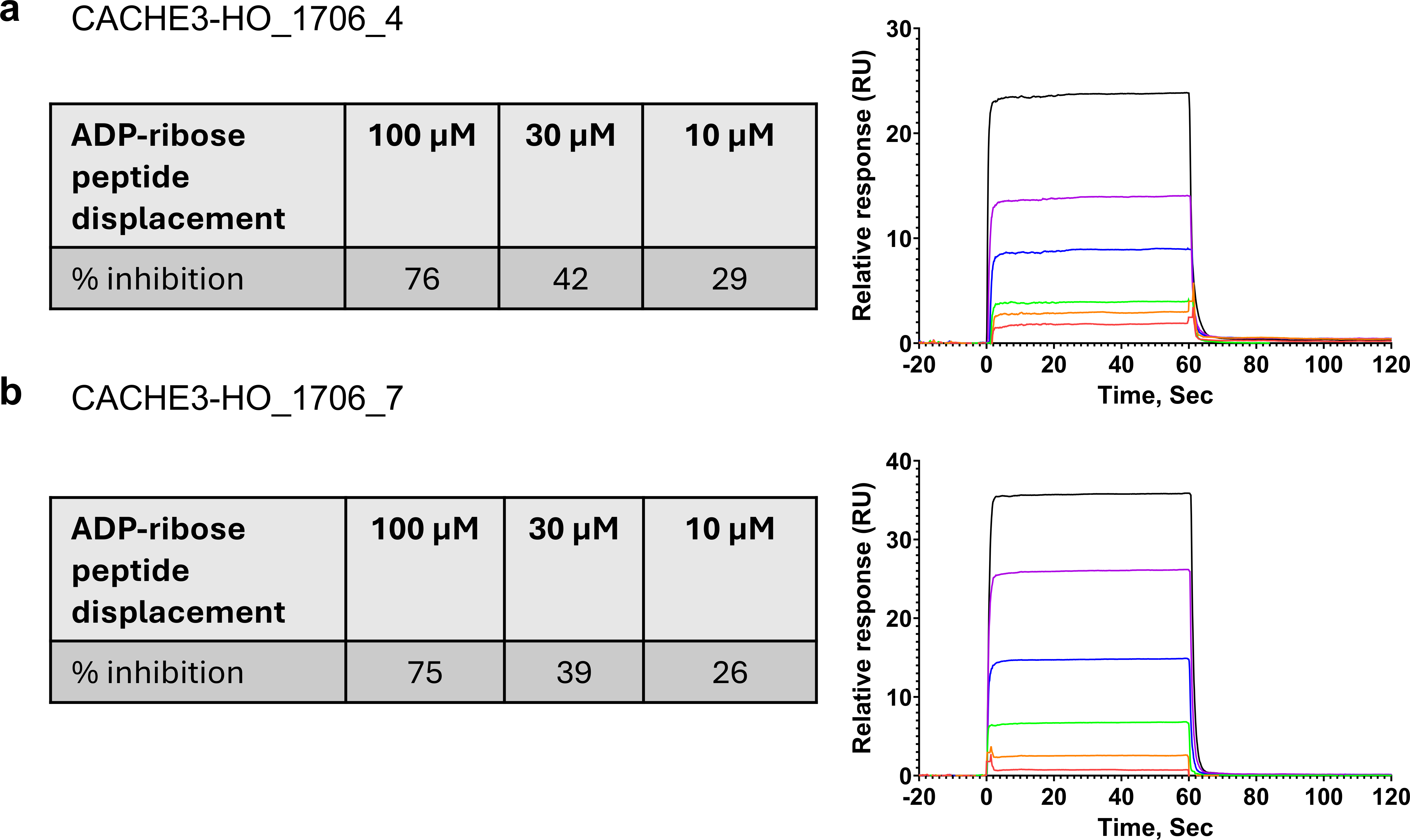
**

***Supplementary Figure 1.*** *ADP-ribose peptide displacement and SPR ligand binding confirmation for a) CACHE3-HO_1706_4 and c) CACHE3-HO _1706_7.*
